# Small Extracellular Vesicles (sEVs) Biogenesis Molecular Players Are Associated with Clinical Outcome of Colorectal Cancer Patients

**DOI:** 10.3390/cancers15061685

**Published:** 2023-03-09

**Authors:** Anastasia Kottorou, Foteinos-Ioannis Dimitrakopoulos, Georgia Diamantopoulou, Foteini Kalofonou, Michalis Stavropoulos, Konstantinos Thomopoulos, Thomas Makatsoris, Angelos Koutras, Haralabos Kalofonos

**Affiliations:** 1 Division of Oncology, Department of Medicine, University Hospital of Patras, 26504 Rio, Greece; 2 Molecular Oncology Laboratory, Medical School, University of Patras, 26504 Rio, Greece; 3 Division of Gastroenterology, University Hospital of Patras, 26504 Rio, Greece; 4 Department of Oncology, The Royal Marsden NHS Foundation Trust, London SW3 6JJ, UK; 5 Department of Surgery, Medical School, University of Patras, 26504 Rio, Greece

**Keywords:** colorectal cancer, sEVs, exosomes, survival, expression

## Abstract

**Simple Summary:**

Numerous studies have emerged into the role of small extracellular vesicles (sEVs), including exosomes in cancer and colorectal cancer in proliferation, metastasis, epithelial-to-mesenchymal transition, angiogenesis and tumor microenvironment. The available data regarding the clinical significance of gene expression of molecules, which participate in sEVs biogenesis, are extremely limited. In the present study, we evaluated the expression of the most important genes, which are implicated in sEVs biogenesis, and their association with sEVs plasma levels as well as with the clinical outcome of patients with colorectal cancer.

**Abstract:**

A growing number of studies have shed light on the role of small extracellular vesicles (sEVs), including exosomes, in colorectal cancer (CRC). Available data regarding the clinical significance of molecular players in CRC, implicated in sEVs biogenesis, is limited. In this study, we assessed the expression of the most important genes which are implicated in sEVs biogenesis and their association with sEVs plasma levels, investigated with a double sandwich ELISA assay, as well as with the clinical outcome of patients with CRC. Our study shows that *RAB27A*, *RAB27B*, *RAB2B,* and *RAB3B* mRNA levels were lower in tumor tissues compared to tumor adjacent, non-malignant tissues (*p* < 0.001, *p* = 0.009, *p* = 0.011, and *p* < 0.001, respectively). In addition, high tumor expression of *RAB27A*, *RAB27B*, *RAB9A*, *RAB11B,* and *STX1A* was favorable of a 5-year survival (*p* = 0.038, *p* = 0.015, *p* = 0.008, *p* = 0.002, and *p* = 0.028, respectively). Furthermore, patients with adenomas had lower overall plasma sEVs concentrations, compared to healthy volunteers (*p* = 0.026), while no statistically significant differences were observed in the overall or tumor-derived plasma sEVs concentration (*p* = 0.885 and *p* = 0.330, respectively) of CRC patients. In conclusion, sEVs biogenesis has a potentially significant role in CRC, with *RAB27A*, *RAB27B*, *RAB9A*, *RAB11B*, and *STX1A* having a promising role in survival outcomes.

## 1. Introduction

During the last decade, an increasing number of studies have emerged into the role of small extracellular vesicles (EVs), including exosomes in cancer and colorectal cancer (CRC) in particular. SEVs are small spherical EVs (30–150 nm) which are enclosed in a double lipid membrane. Their cargo can consist of DNA, mRNAs, microRNAs (miRNAs), long non-coding RNAs (lncRNAs), and proteins [1,2,3,4]. Initially, sEVs were considered to be products of metabolism. However, it has now become clear that sEVs play an important role in many functions, including intercellular communication, antigen presentation, and transportation of important biological factors [5].

Numerous studies have documented that cancer cells produce more sEVs in vitro as well as in vivo compared to non-malignant cells. Cancer-derived sEVs seem to activate proliferation and angiogenesis pathways and play a significant role in tumor invasion as well as in immune suppression [6,7]. Furthermore, a growing number of studies have also highlighted the role of sEVs in cancer progression, modification of the tumor microenvironment, pre-metastatic niche formation, metastasis as well as resistance to therapy [5].

The origin of the sEVs is intracellular and their biogenesis is completed in three stages. In the first stage of their biogenesis, an early endosome is formed. Next, the early endosome matures into a late endosome, which gives rise to a multi-vesicular body (MVBs) with intraluminal vesicles (ILVs) inside their cavity. These multivesicular bodies can either be degraded in the lysosomes or fused with the cell membrane, resulting in subsequent release of the intraluminal vesicles as sEVs [8,9]. This multistep process of sEVs biogenesis involves the interaction between many proteins or protein complexes and remains to be fully elucidated. In brief, the endosomal sorting complex required for transport (ESCRT) has a pivotal role in the formation of ILVs as well as in the protein cargo sorting [10]. However, ESCRT-independent mechanisms have also been described (they have been reviewed here [11]). Additionally, numerous molecules have been documented to participate in MVBs formation, selective cargo sorting into ILVs and sEVs release, including Rab GTPases and syntaxins, which are components of SNARE complexes (recently reviewed here [12]).

Focusing on CRC, numerous studies have supported the involvement of sEVs in proliferation, metastasis, EMT (epithelial-to-mesenchymal transition), and angiogenesis in CRC as well as the tumor microenvironment [13]. In addition, sEVs have been associated with treatment resistance [14] and, interestingly, they have also been used as useful delivery platforms for CRC treatment [15,16]. Although the published evidence supports the role of sEVs on the initiation and progression of CRC, the available data regarding the clinical significance of gene expression of molecules, which participate in sEVs biogenesis, are extremely limited. In this study, we evaluated the expression of the most important genes, which are implicated in sEVs biogenesis, and their association with sEVs plasma levels as well as with the clinical outcome of patients with CRC.

## 2. Materials and Methods

### 2.1. Gene Selection

For the selection of genes with the highest impact in exosome biogenesis, especially in CRC, the publicly available literature was carefully reviewed (via Pubmed and Google Scholar) using as criteria the names of genes implicated in sEVs biogenesis as well as the cancer type (CRC) leading then to 9 relevant studies. In addition, we used publicly available data from TCGA (The Cancer Genome Atlas) and Genotype-Tissue Expression (GTEx) projects, using the web server GEPIA (Gene Expression Profiling Interactive Analysis) for cancer and normal gene expression profiling [17]. Finally, 8 genes with differential expression between CRC and normal tissues were selected, since they were considered as the most promising (Supplementary Figure S1). In addition, and in order to evaluate the impact of each candidate gene in survival outcome, we used the aforementioned web server GEPIA [17] as well as the internet-based tool “KMplotter”, focusing on patients with CRC (Supplementary Figures S2 and S3) [18]. The 8 genes, which were finally selected as the most promising, were *RAB2B (Ras-Related Protein Rab-2B)*, *RAB3B (Ras-Related Protein Rab-3B)*, *RAB9A (Ras-Related Protein Rab-9A)*, *RAB11B (Ras-Related Protein Rab-11B)*, *RAB27A (Ras-Related Protein Rab-27A)*, *RAB27B (Ras-Related Protein Rab-28B)*, *STX1A (Syntaxin 1A)*, and *VAMP7 (Vesicle-Associated Membrane Protein 7)*.

### 2.2. Patients and Samples

This is a prospective study which was approved by the Scientific Committee and the Committee on Research and Ethics of the University Hospital of Patras, Greece (451/30/9/2016) and informed consent was obtained from all participants. In order to investigate our hypothesis, a total of 121 CRC patients, 39 patients with adenomas, and 39 healthy volunteers were enrolled in this study. Clinicopathological parameters of the CRC patients and demographic characteristics of all participants are shown in Table 1. All participants of the study were medically managed at the University Hospital of Patras (Departments of Gastroenterology, Surgery and Oncology). Tumor and non-malignant paired tissues were obtained from 109 CRC patients and stored in RNAlater RNA Stabilization Reagent (Sigma–Aldrich, St. Louis, MO, USA) at −80 °C. Diagnosis was determined based on the histological assessment. Blood samples were also collected from 65 CRC patients, all the adenomas patients as well as all the healthy volunteers by phlebotomy prior to colonoscopy or prior to surgical excision of the tumor or postoperatively. All blood samples were collected in K_2_EDTA Vacuette tubes (Greiner BioOne, Frickenhausen, Germany). Plasma was prepared within 2 h of blood collection by centrifugation at 1500× *g* for 20 min at 4 °C and was stored at −80 °C until further processing.

### 2.3. RNA Isolation and cDNA Synthesis

RNA was extracted from 109 CRC and 60 tumor adjacent and paired non-malignant tissue specimens using the PerfectPure RNA Tissue Kit (5Prime, Hamburg, Germany), according to the manufacturers’ instructions. RNA samples were also incubated with DNase (Ambion, Austin, TX, USA), quantified using a Nanodrop-1000 spectrophotometer (NanoDrop, Fisher Thermo, Wilmington, DE, USA) and then stored at −80 °C. Four μg of RNA were reverse transcribed into cDNA using 100 U of Superscript III Reverse Transcriptase (Life Technologies), 300 ng of random primers (Foundation for Research and Technology-Hellas, Crete, Greece) and 5 nM dNTPs (Enzyquest, Crete, Greece) in a total volume of 50 μL. A no enzyme control was used to ensure that the RNA samples were DNA-free. The mixture was incubated in a C1000 Touch thermal cycler (Bio-Rad) at 25 °C for 5 min, 50 °C for 60 min, and 70 °C for 15 min. cDNA was diluted to 25 ng/μL and stored at −20 °C.

### 2.4. Gene Expression Quantification

Quantification of *RAB2B*, *RAB3B*, *RAB9A*, *RAB11B*, *RAB27A*, *RAB27B*, *STX1A*, and *VAMP7* gene expression was performed by Quantitative Real Time PCR (qRT-PCR) with primers and probes designed by our group. The used primers and probes were synthesized by Metabion International AG (Martinsried, Germany) and sequences are shown in Table 2. The qPCR reactions were carried out in triplicate, in a total volume of 20 μL, containing 3 μL of cDNA, 300 nM sense primer, 300 nM anti-sense primer, 100 nM probe, 10 nM reference dye (ThermoFischer Scientific, Waltham, MA, USA), 200 μM dNTPs (ThermoFischer Scientific, Waltham, MA), and a single unit of Platinum Taq DNA Polymerase (ThermoFischer Scientific, Waltham, MA, USA) in 1× buffer containing 16.6 mM ammonium sulfate, 67 mM Tris pH 8.8, 6.7 mM MgCl_2_ and 10 mM 2-mercaptoethanol (Herman et al. 1996). Cycling conditions were as follows: 95 °C for 10 min and 45 cycles at 95 °C for 30 s and at 60 °C for 1 min, 72 °C for 30 s within the StepOne Plus (ThermoFischer Scientific, Waltham, MA, USA). Relative gene expression levels were calculated using the LinRegPCR software [19]. Gene expression levels were finally normalized to the levels of *IPO8 (Importin 8) gene*, which was used as a reference gene [20]. Previously designed by our group primers for *IPO8* were used [21]. Blind experimental design and analysis was performed with respect to the specimen’s type and participants’ identities and data.

### 2.5. Plasma sEVs Quantification

Overall plasma sEVs levels were determined for 65 CRC patients, 39 adenoma patients, and 39 healthy subjects using ExoTest double sandwich ELISA quantification kit for overall sEVs (HansaBioMed Life Sciences, Tallinn, Esthonia) which is based on CD9 exosome marker, according to the manufacturer’s instructions as described before [22]. Moreover, tumor-derived plasma sEVs concentration was determined for the same CRC patients, using ExoTest quantification kit for tumor-derived exosomes (HansaBioMed Life Sciences, Tallinn, Esthonia). The Exostest for quantification of Tumor-derived vesicles allows an enrichment of EVs from tumor source, using an anti-TM9SF4 antibody, which is highly expressed in tumor tissues and derived EVs, and HansaBioMed has patented as tumor marker, while the detection is performed with CD9. Briefly, 500 μL plasma were used from each participant and was centrifuged 3 times. Samples were analyzed in duplicate, with 100 μL being used in each well of the 96-well ELISA plate and overnight incubated for 15 h in 4 °C. A substrate chromogen was applied for 5 min in the final step. A standard curve was used for exosomes concentration absolute quantification. Verification of the isolated exosomes with these kits has been performed using transmission electron microscopy (TEM) and has previously been presented [22].

### 2.6. Statistical Analysis

IBM SPSS Statistics for Windows, Version 21.0 (Armonk, NY, USA: IBMCorp.) was used for all the analyses performed. Intergroup comparisons for the association of gene expression levels between cancer and non-cancerous tissues were performed using Kruskal–Wallis non-parametric test. Comparisons between related groups were performed using Wilcoxon paired samples test for expression levels of cancer and non-cancerous tissues and gene expression and sEVs levels of the same patient. Mann–Whitney and Kruskal–Wallis non-parametric tests were used for the association of gene expression levels with clinicopathological characteristics of the patients. Pearson correlation coefficient was used among all genes expression and sEVs concentration. The Kaplan–Meier curves and the log rank test were used for the estimation of survival rates and the prognostic significance of gene expression and sEVs concentration was evaluated by Cox regression analysis. Overall, survival (OS) of the CRC patients was assessed after a follow-up period of 60 months by using past medical histories or through direct personal contact (via phone or in person). For all comparisons, statistical significance was defined as *p* < 0.05.

## 3. Results

### 3.1. Lower Gene Expression in Tumor vs. Normal

**Figure 1:**
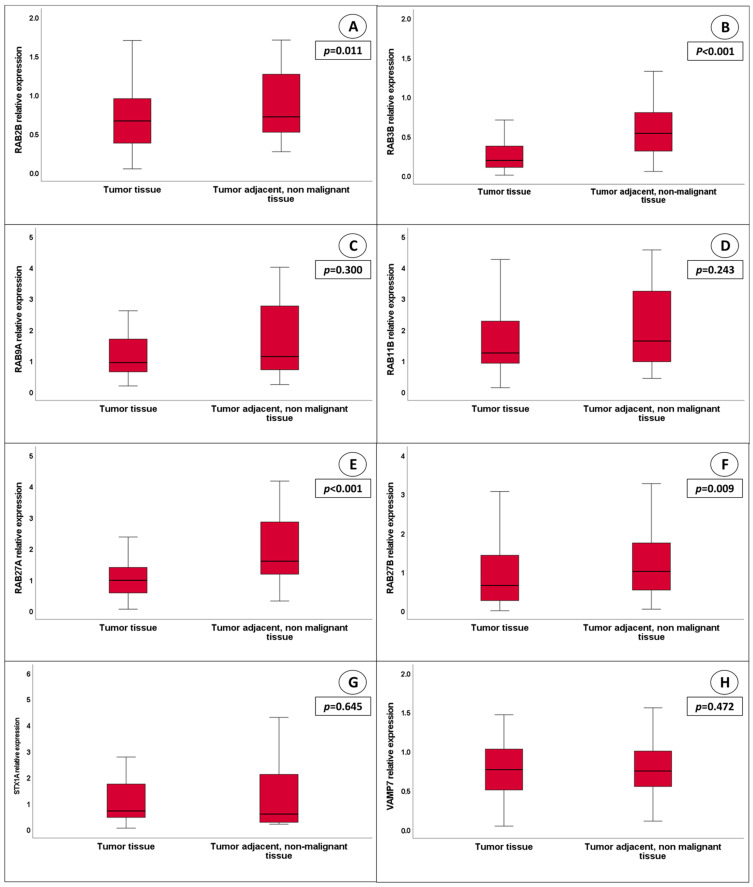
Relative gene expression between tumor (n = 109) and tumor-adjacent (n = 60) tissues of *RAB2B* (**A**), *RAB3B* (**B**), *RAB9A* (**C**), *RAB11B* (**D**), *RAB27A* (**E**), *RAB27B* (**F**), *STX1A* (**G**) and *VAMP7* (**H**) in our CRC patients’ cohort. Gene expression of *RAB2B*, *RAB3B*, *RAB27A,* and *RAB27B* is lower in tumor tissues compared to tumor adjacent, non-malignant tissues, while for the other genes no statistically significant differences are observed.

*RAB2B*, *RAB3B*, *RAB9A*, *RAB11B*, *RAB27A*, *RAB27B*, *STX1A*, and *VAMP7* gene expression was quantified in 109 CRC and 60 tumor adjacent and paired non-malignant tissue specimens. Interestingly, *RAB27A*, *RAB27B*, *RAB2B*, *RAB3B* mRNA levels were lower in tumor tissues compared to tumor adjacent, non-malignant tissues (*p* < 0.001, *p* = 0.009, *p* = 0.011 and *p* < 0.001, respectively, Figure 1). On the other hand, no statistically significant differences were observed between cancerous and tumor-adjacent tissues for *RAB9A*, *RAB11B*, *STX1A*, and *VAMP7* (*p* = 0.300, *p* = 0.243, *p* = 0.646, and *p* = 0.472, respectively).

### 3.2. Association of Gene Expression with Clinicopathological Parameters of the Patients

**Figure 2:**
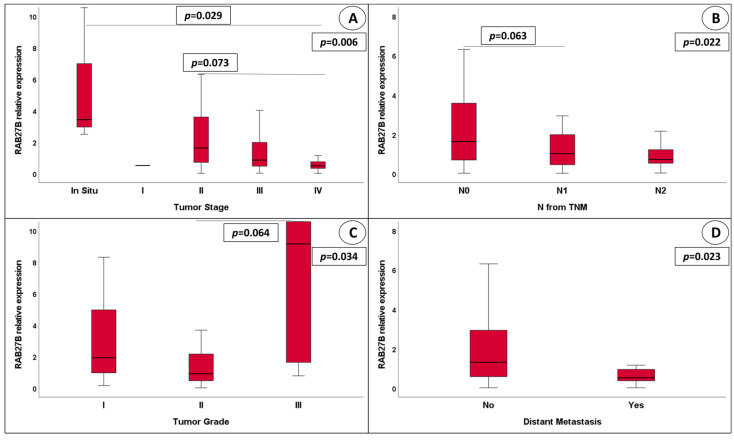
Relative gene expression of (**A**) *RAB27B* with regards to disease stage, (**B**) *RAB27B* with regards to lymph node infiltration, (**C**) *RAB27B* with regards to tumor grade and (**D**) *RAB27B* with regards to metastatic status in our CRC patients’ cohort (n = 109). Relative *RAB27B* expression is decreasing as disease stage increases, with tumor with lymph node infiltration and distant metastases having lower *RAB27B* expression. *RAB27B* expression is higher in grade III tumors compared to grade II tumors.

When analyzed with regard to clinicopathological parameters, *RAB27B* mRNA expression showed significant association with tumor stage. More specifically, *RAB27B* expression was gradually decreased as stage was increased, with the highest expression being observed in *in situ* tumors and the lowest being observed in stage IV tumors (*p* = 0.006, Figure 2A). Similarly, *RAB27B* expression was associated with N status, with expression being gradually decreased from N0 status (without lymph nodes infiltration) to N2 status (tumor infiltration in more than 4 regional lymph nodes) (*p* = 0.022, Figure 2B). Regarding tumor grade, higher *RAB27B* expression was observed in grade III tumors, compared to grade I and grade II tumors (*p* = 0.023, Figure 2C). Moreover, *RAB27B* expression was higher in tumors without distant metastasis compared to those with metastases (*p* = 0.034, Figure 2D). Among all other genes no statistically significant difference was observed in their expression and tumor stage or tumor grade or N status (Supplementary Figures S4–S6).

**Figure 3:**
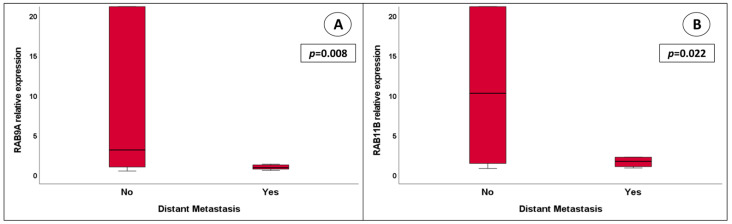
Relative gene expression of *RAB9A* (**A**) and *RAB11B* (**B**) with regards to the presence of distant metastases (n = 109). Patients with distant metastases have lower tumor expression of *RAB9A* and *RAB11B*.

**Figure 4:**
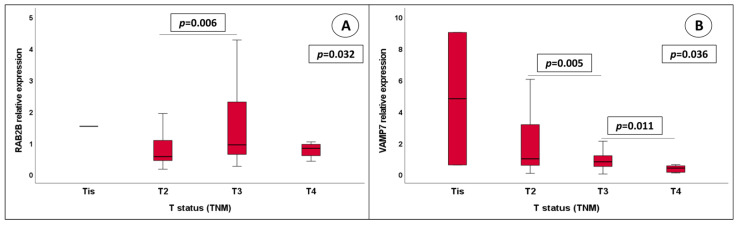
Relative gene expression of *RAB2B* (**A**) and *VAMP7* (**B**) with regards to T status (n = 109). Higher *RAB2B* relative expression is observed in T3 tumors compared to T2. *VAMP7* relative expression is decreasing as T status increases, with T3 tumors having lower expression than T2 tumors and T4 tumors having lower expression than T3 tumors.

Among the other studied genes, *RAB9A* and *RAB11B* were associated with distant metastasis. In particular, lower *RAB9A* and *RAB11B* expression was observed in tumors with distant metastases (*p* = 0.008 and *p* = 0.022, Figure 3A,B, respectively). Expression of all genes was also analyzed in regard to TNM. TNM staging system is a tumor classification system in which T describes the size of the tumor and any spread into nearby tissue, N describes infiltration of nearby lymph nodes, and M describes presence of distant metastasis. In regards to T status from TNM staging system, *RAB2B* expression differed among different T status (*p* = 0.032, Figure 4A), as well as *VAMP7* expression was gradually decreased as T was increasing (*p* = 0.036, Figure 4B). Among all other genes no statistically significant difference was observed in their expression and distant metastasis or T status (Supplementary Figures S7 and S8).

### 3.3. High Gene Expression Favorable for Survival Outcomes

**Figure 5:**
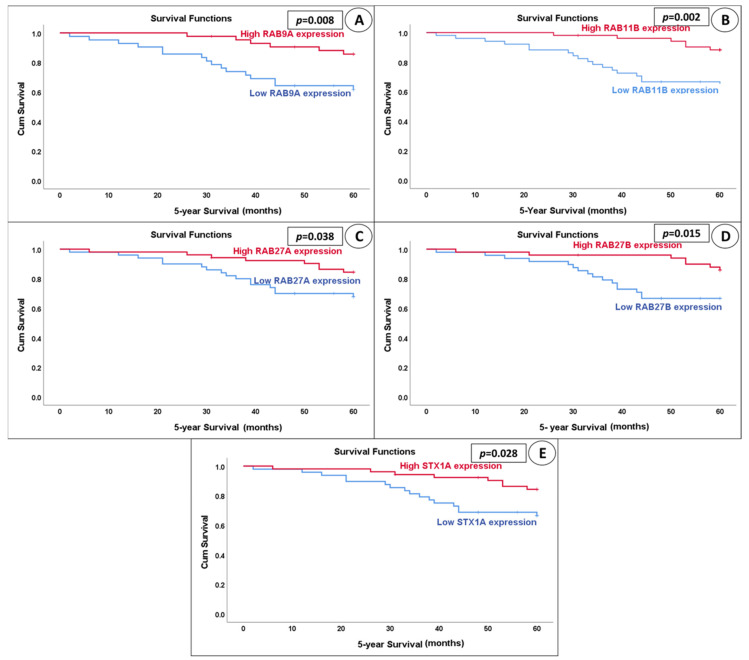
Kaplan Meier curves for 5-year overall survival of *RAB27A* (**A**), *RAB27B* (**B**), *RAB9A* (**C**), *RAB11B* (**D**), and *STX1A* (**E**) (n = 109). Patients with higher expression of those genes have longer 5-year survival.

Interestingly, when gene expression was analyzed in terms of patients’ survival, expression of five out of the eight studied genes was associated with a 5-year survival, following the same pattern. More specifically, high tumor expression of *RAB27A*, *RAB27B*, *RAB9A*, *RAB11B,* and *STX1A* was favorable of a 5-year survival (*p* = 0.038, *p* = 0.015, *p* = 0.008, *p* = 0.002, and *p* = 0.028, respectively, Figure 5), while no association was observed with the rest of the genes (Supplementary Figure S9).

### 3.4. Quantification of Overall and Tumor Plasma sEVs Concentration

**Figure 6:**
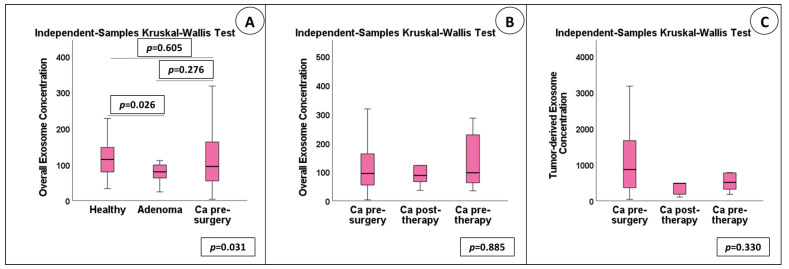
Overall plasma sEVs concentration in healthy controls (n = 39), patients with adenomas (n = 39) and patients with CRC (n = 65) (**A**), overall plasma sEVs concentration in patients with CRC before surgery (n = 65), before (n = 7) and post systematic treatment (n = 5) (**B**) and tumor-derived plasma sEVs concentration in patients with CRC before surgery (n = 65), before (n = 7) and post systematic treatment (n = 5) (**C**).

We next sought to explore the plasma sEVs concentration in patients with CRC, with colorectal adenomas as well as of healthy volunteers. In total, overall plasma sEVs concentration was evaluated in 65 patients with CRC, before having surgery, 39 patients with colorectal adenoma, and 39 healthy volunteers. Interestingly, patients with adenomas had lower overall plasma sEVs concentrations, when compared to healthy volunteers (*p* = 0.026, Figure 6A). On the other hand, there were no significant differences in overall plasma sEVs levels between cancer patients and healthy controls or adenoma patients (*p* = 0.276 and *p* = 0.605, respectively).

In addition, we compared overall and tumor-derived plasma sEVs concentration between the 65 patients with CRC before having surgery with a small cohort of 7 patients with metastatic colorectal adenocarcinoma before systematic therapy as well as with 5 patients with metastatic colorectal adenocarcinoma after systematic therapy. No statistically significant differences were observed for neither the overall nor the tumor-derived plasma sEVs concentration (*p* = 0.885 and *p* = 0.330, Figure 6B,C, respectively).

### 3.5. Association of Plasma sEVs Concentration with Survival Outcome

**Figure 7:**
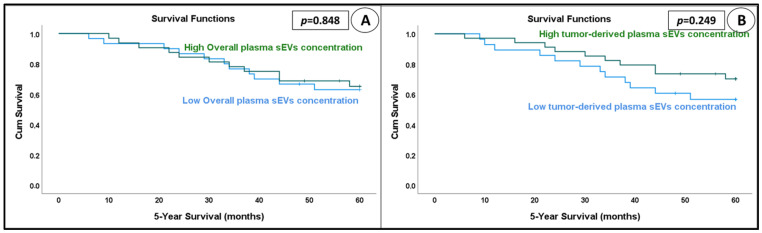
Kaplan Meier curves for 5-year overall survival of overall sEVs plasma concentrations (**A**) and of tumor-derived plasma sEVs concentrations (**B**) using median as cutoff point (n = 65). No statistically significant differences were observed between 5-year survival and plasma sEVs concentration, although there was a trend for patients with higher tumor-derived plasma sEVs concentration having longer survival.

When overall plasma sEVs concentration of CRC patients before having surgery was analyzed in terms of patients’ 5-year survival, no statistically significant difference was observed using median as cut-off point (*p* = 0.848, Figure 7A). However, when the same analysis was performed for tumor-derived plasma sEVs concentration of the same patients, there was a trend of survival benefit for patients with higher tumor-derived plasma sEVs concentration, although it did not reach statistical significance (HR = 0.594, *p* = 0.249, Figure 7B).

### 3.6. Correlations of SEVs Concentrations with Gene Expression

All correlations as well as coefficients and *p* values are shown in Table 3. The correlation between overall and tumor-derived plasma sEVs concentrations in CRC patients before having surgery revealed a moderate positive correlation (r = 0.408, *p* < 0.001, Table 3). Moreover, a moderate positive correlation was also observed between *RAB11B* expression and overall plasma sEVs concentration (r = 0.318, *p* = 0.045, Table 3). Additionally, significantly strong positive correlations were also observed between gene expression of the studied markers, with the strongest correlations found between gene expression of *RAB2B* and *RAB27B* as well as between *RAB9A* and *RAB11B* (r = 0.912, *p* < 0.001, and r = 0.812, *p* < 0.001, respectively, Table 3).

## 4. Discussion

The significant role of sEVs in CRC initiation and progression has been confirmed with numerous published studies. Many studies have shed light on the mechanisms through which sEVs are implicated in CRC development and progression [23]. However, limited data are available, regarding the clinical significance of key molecules related to the exosomal biogenesis. In this context, we assessed the clinical value of 8 genes (*RAB2B*, *RAB3B*, *RAB9A*, *RAB11B*, *RAB27A*, *RAB27B*, *STX1A*, and *VAMP7),* which are implicated in sEVs biogenesis, as well as their association with overall and tumor-derived plasma sEVs levels.

One of the most interesting findings of this study was that mRNA expression of *RAB27B* was lower in tumor tissues compared to tumor adjacent non-malignant tissues. Moreover, *RAB27B* expression was associated with stage, lymph node infiltration, and distant metastasis, with higher expression demonstrated in tumors of earlier stages, without lymph node infiltration or distant metastasis. Interestingly, higher *RAB27B* expression was also favorable of a 5-year survival. Our findings are in agreement with those of the bioinformatic analysis we performed (Supplementary Figures S2 and S3), as well as with those of Dong et al., who have reported that RAB27B expression is lower in CRC compared to nonmalignant tissue samples and is a favorable prognostic factor [24]. On the contrary, Bao et al. have reported that *RAB27B* expression is higher in CRC tissues than non-cancerous tissues and is associated with lymph nodes infiltration, distant metastasis, and worse overall survival [25]. Similarly, based on TCGA data analysis, Hua et al. have shown that RAB27B is possibly implicated in rectal adenocarcinoma metastasis [26], while Cheng et al. have shown that RAB27B plays a significant role in the secretion of CRC stem cell sEVs, which seem to promote cancer initiation forming an immunosuppressive tumor microenvironment [27].

Interestingly, we observed that mRNA expression levels of *RAB27A* were lower in CRC tissues compared to tumor adjacent tissues, with higher expression having favorable prognostic value. In line with our results, Dong et al. also have reported that RAB27A expression is lower in CRC compared to non-malignant tissue samples, while negative protein RAB27A expression was associated with distant metastasis, local recurrence, and worse survival [24]. Similarly, another study by Shi et al. also suggested that high *RAB27A* expression is a favorable prognostic factor for CRC patients [28]. However, in the same study, the authors reported that CRC tissues have higher *RAB27A* expression than non-cancerous tissues [28]. On the other hand, our results are also confirmed by TCGA data analysis we performed (Supplementary Figure S1). In addition, although we did not observe any statistically significant association between *RAB27A* mRNA and plasma sEVs concentrations, however, it seems that *RAB27A* plays a pivotal role in sEVs biogenesis in CRC. Huang and Feng have shown that silencing of *RAB27A* in hypoxic CRC cells results in suppression of exosomes secretion and inhibition of proliferation and migration of endothelial cells [29]. In addition, *RAB27A* affects CRC initiation and progression not only through exosome formation, but also through induction of stemness of CRC cells via NF-κB signaling [30]. This association, between RAB27A and NF-κB signaling, provides an alternative to sEVs mechanism through which RAB27A could influence clinical outcome of CRC patients.

Intriguing was also the finding that higher tumor expression levels of some of the studied genes (*RAB27A*, *RAB27B*, *RAB9A*, *RAB11B*, and *STX1A*) was indicative of a favorable 5-year survival, an observation which was compatible with the findings derived from the analysis of TCGA data presented in the “Gene selection” paragraph. Higher expression of the key molecules of sEVs biogenesis may lead to higher sEVs production [31]. In our cohort, this potent association with plasma sEVs was not observed with the exception of *RAB11B*, which was correlated with plasma sEVs concentration. Plasma exosomes are a pool of sEVs derived from different cells. However, it is known that tumor-derived sEVs have the potential to function mainly locally reshaping the tumor microenvironment towards to a more immunosuppressive state, by remodeling the extracellular matrix and promoting tumor cell metabolism, growth, and metastasis [23]. Therefore, this lack of correlation between gene expression and tumor-derived plasma exosome concentrations may reflects the mechanism through which cancer-derived sEVs affect CRC (mainly locally) and not the absence of impact in pathophysiological level.

In our study, adenoma patients had lower overall plasma sEVs levels compared to healthy controls and CRC patients. According to our knowledge, the relevant published studies, focusing on plasma sEVs levels in healthy controls, patients with colon adenomas and CRC, are limited. In 2018, Kobayashi et al. supported that CRC patients had higher plasma sEVs levels compared to healthy controls or hyperplastic polyps or low grade adenomas [32]. In particular, in this study, 5 participants with adenocarcinoma (4 pTis and 1 pT1), 8 with high-grade adenoma, 4 with low-grade adenoma, 4 with hyperplastic polyps, and 4 healthy controls were enrolled. However, the participants in that study was not adequate in order to demonstrate this difference, since included patients with adenocarcinoma (n = 5) had mainly a non-infiltrative stage (Tis).

Despite the interesting results of the study, we have to acknowledge that there are some limitations in the current study. First of all, a limitation of our study is the number of the participants. Additionally, it would be desirable and more informative if a two-phase design has been followed, however, due to the number of the enrolled patients this approach was not possible. Furthermore, stage I and IV CRC patients are not equally represented in our cohort. Moreover, heterogeneity of the sub cohorts with regards to age could be another important issue. Another limitation of this study is that for the isolation and quantification of plasma exosomes a test based only on the CD9 expression was used.

## 5. Conclusions

In conclusion, our findings suggest that exosome biogenesis pathway has a potential significant role in CRC, with *RAB27A*, *RAB27B*, *RAB9A*, *RAB11B*, and *STX1A* being the most promising markers, regarding their impact in survival outcome. In addition, although this study does not confirm a statistically significant association between the exosome pathway effectors and plasma sEVs, however, it seems that these molecules may affect mainly tumor-derived plasma sEVs. More studies are needed to further clarify this association and their potential clinical value.

## Figures and Tables

**Table 1:** Demographic and clinicopathological characteristics of colorectal cancer/adenoma patients and healthy controls. Abbreviations: N/A, not available.

Demographic/Clinicopathological Characteristics		Cancer Patients	Adenoma Patients	Healthy Individuals
**Gender**	**Male**	72 (59.5%)	26 (66.6%)	13 (33.3%)
	**Female**	49 (40.5%)	13 (33.3%)	26 (66.6%)
**Age Group**	**66≥**	40 (33.1%)	26 (66.6%)	24 (61.5%)
	**>66**	81 (66.9%)	13 (33.3%)	15 (39.5%)
**Stage**	**In situ**	7 (5.8%)	-	-
	**I**	1 (0.8%)	-	-
	**II**	58 (47.9%)	-	-
	**III**	41 (33.9%)	-	-
	**IV**	8 (6.6%)	-	-
	**N/A**	6 (5%)	-	-
**Grade**	**I**	15 (12.4%)	-	-
	**II**	80 (66.1%)	-	-
	**III**	5 (4.1%)	-	-
	**N/A**	21 (17.4%)	-	-
**Primary Site**	**Right Colon**	42 (34.7%)	-	-
	**Left Colon and Sigmoid**	34 (28.1%)	-	-
	**Rectum**	39 (32.2%)	-	-
	**N/A**	6 (5%)	-	-
**Lymph Node metastasis**	**No**	68 (56.2%)	-	-
	**Yes**	45 (37.2%)	-	-
	**N/A**	8 (6.6%)	-	-
**Distant metastasis**	**No**	98 (81%)	-	-
	**Yes**	9 (7.4%)	-	-
	**N/A**	14 (11.6%)	-	-

**Table 2:** The list of primers and probes used for gene expression analysis by quantitative Real Time PCR.

Gene	Forward 5′–3′	Reverse 5′–3′	Probe 5′	Size (bp)
RAB2B	CAAATCTGGGATACGGCTG	GTACACCAGCAG TGCTC	FAM/TCCTTC CGTTCTATCACCCGT/BHQ	87
RAB3B	ACGAGAAGCGGGTGAAAC	TAATAGGCTGTTGTGATGGTC	FAM/CTGGGACACAGCTGGGCA/BHQ	76
RAB9A	TCTCTCTGTCCTCATTGC	CTCAAAAGCTTCAAGAACCC	FAM/TCGCGGCCACACGAAAGA/BHQ	89
RAB11B	TTCAAAGTGGTGCTCATCG	TCCAGGTTGAACTCGTTG	FAM/AGGCGTGGGCAAGAGCAA/BHQ	83
RAB27A	GCACTCGCAGAGAAATATGG	TGCTTGGCTTATGTTTGTCC	FAM/CCCTACTTTGAAACTAGTGCTGCCA/BHQ	72
RAB27B	ACCAGTCAACAGAGCTTC	ATATCTGGATTTTCACAATAAGC	FAM/GAAACTGGATGAGCCAACTGCA/BHQ	80
VAMP7	AACTACCAGCAGAAATCTTG	ATGAACACAATTGATACGATG	FAM/AGCCATGTGTATGAAGAACCTCAA/BHQ	87
STX1A	CATTGACAAGATCGCAGAG	CTCCTCCTTCGTCTTCTC	FAM/GAGGAGGTGAAGCGGAAGCA/BHQ	94

**Table 3:** Correlation coefficients and *p* values among sEVs concentrations and genes expression levels.

Correlations											
		Overall Plasma sEVs Levels	Tumor-Derived Plasma sEVs Levels	RAB2B Expression	RAB3B Expression	RAB9A Expression	RAB11B Expression	RAB27A Expression	RAB27B Expression	VAMP7 Expression	STX1A Expression
**Overall plasma sEVs levels**	Pearson Correlation	1	0.408 **	0.265	−0.100	0.291	0.318 *	0.041	−0.048	0.180	0.210
	Sig. (2-tailed)		**<0.001**	0.103	0.541	0.069	0.045	0.800	0.770	0.267	0.213
**Tumor-derived plasma sEVs levels**	Pearson Correlation	0.408 **	**1**	0.052	−0.059	0.047	0.019	−0.014	0.017	0.107	0.033
	Sig. (2-tailed)	**<0.001**		0.753	0.719	0.772	0.905	0.931	0.918	0.511	0.848
**RAB2B expression**	Pearson Correlation	0.265	**0.052**	1	0.483 **	0.799 **	0.456 **	0.438 **	0.231 *	0.229 *	0.537 **
	Sig. (2-tailed)	0.103	**0.753**		**<0.001**	**<0.001**	**<0.001**	**<0.001**	**0.026**	**0.026**	**<0.001**
**RAB3B expression**	Pearson Correlation	−0.100	**−0.059**	0.483 **	1	0.718 **	0.597 **	0.663 **	0.527 **	0.298 **	0.310 **
	Sig. (2-tailed)	0.541	**0.719**	**<0.001**		**<0.001**	**<0.001**	**<0.001**	**<0.001**	**0.004**	**0.002**
**RAB9A expression**	Pearson Correlation	0.291	**0.047**	0.799 **	0.718 **	1	0.812 **	0.570 **	0.294 **	0.171	0.350 **
	Sig. (2-tailed)	0.069	**0.772**	**<0.001**	**<0.001**		**<0.001**	**<0.001**	**0.006**	0.114	**<0.001**
**RAB11B expression**	Pearson Correlation	0.318 *	**0.019**	0.456 **	0.597 **	0.812 **	1	0.578 **	0.126	0.159	0.615 **
	Sig. (2-tailed)	**0.045**	**0.905**	**<0.001**	**<0.001**	**<0.001**		**<0.001**	0.208	0.109	**<0.001**
**RAB27A expression**	Pearson Correlation	0.041	**−0.014**	0.438 **	0.663 **	0.570 **	0.578 **	1	0.229 *	0.226 *	0.353 **
	Sig. (2-tailed)	0.800	**0.931**	**<0.001**	**<0.001**	**<0.001**	**<0.001**		**0.021**	**0.022**	**<0.001**
**RAB27B expression**	Pearson Correlation	−0.048	**0.017**	0.231 *	0.527 **	0.294 **	0.126	0.229 *	1	0.397 **	0.001
	Sig. (2-tailed)	0.770	**0.918**	**0.026**	**<0.001**	**0.006**	0.208	**0.021**		**<0.001**	0.992
**VAMP7 expression**	Pearson Correlation	0.180	**0.107**	0.229 *	0.298 **	0.171	0.159	0.226 *	0.397 **	1	0.223 *
	Sig. (2-tailed)	0.267	**0.511**	**0.026**	**0.004**	0.114	0.109	**0.022**	**<0.001**		**0.027**
**STX1A expression**	Pearson Correlation	0.210	**0.033**	0.537 **	0.310 **	0.350 **	0.615 **	0.353 **	0.001	0.223 *	1
	Sig. (2-tailed)	0.213	**0.848**	**<0.001**	**0.002**	**<0.001**	**<0.001**	**<0.001**	0.992	**0.027**	

** Correlation is significant at the 0.01 level (2-tailed). *. Correlation is significant at the 0.05 level (2-tailed).

## Data Availability

Data is available upon request.

## Supplementary Materials

The following supporting information can be downloaded at: https://www.mdpi.com/article/10.3390/cancers15061685/s1, Figure S1. Dot-plots with superimposed box plots for the expression of 8 selected gene in colorectal cancer (red) and normal tissues (grey) using GEPIA web server. Box plots for the expression of (**A**) *RAB2B*, (**B**) *RAB3B*, (**C**) *RAB9A*, (**D**) *RAB11B*, (**E**) *RAB27A*, (**F**) *RAB27B*, (**G**) *STX1A* and (**H**) *VAMP7A*. Mean and standard deviation values were calculated for the comparison. Abbreviations: COAD, Colon adenocarcinoma; *RAB2B*, Ras-Related Protein Rab-2B; *RAB3B*, Ras-Related Protein Rab-3B; *RAB9A*, Ras-Related Protein Rab-9A; *RAB11B*, Ras-Related Protein Rab-11B; *RAB27A*, Ras-Related Protein Rab-27A; *RAB27B*, Ras-Related Protein Rab-28B; *STX1A*, Syntaxin 1A; *VAMP7*, Vesicle-Associated Membrane Protein 7. Figure S2. Kaplan Meier curves of overall survival using the “KMplotter” for the expression of (**A**) *RAB2B*, (**B**) *RAB3B*, (**C**) *RAB9A*, (**D**) *RAB11B*, (**E**) *RAB27A*, (**F**) *RAB27B*, (**G**) *STX1A* and (**H**) *VAMP7* in colorectal cancer patients using “KMplotter”. Abbrevations: RAB2B, Ras-Related Protein Rab-2B; *RAB3B*, Ras-Related Protein Rab-3B; *RAB9A*, Ras-Related Protein Rab-9A; *RAB11B*, Ras-Related Protein Rab-11B; *RAB27A*, Ras-Related Protein Rab-27A; *RAB27B*, Ras-Related Protein Rab-28B; *STX1A*, Syntaxin 1A; *VAMP7*, Vesicle-Associated Membrane Protein 7. Figure S3. Kaplan Meir curves of overall survival for the expression of (**A**) *RAB2B*, (**B**) *RAB3B*, (**C**) *RAB9A*, (**D**) *RAB11B*, (**E**) *RAB27A*, (**F**) *RAB27B*, (**G**) *STX1A* and (**H**) *VAMP7* in colorectal cancer patients using the “GEPIA”. Abbreviations: *RAB2B*, Ras-Related Protein Rab-2B; *RAB3B*, Ras-Related Protein Rab-3B; *RAB9A*, Ras-Related Protein Rab-9A; *RAB11B*, Ras-Related Protein Rab-11B; *RAB27A*, Ras-Related Protein Rab-27A; *RAB27B*, Ras-Related Protein Rab-28B; *STX1A*, Syntaxin 1A; *VAMP7*, Vesicle-Associated Membrane Protein 7. Figure S4. Relative gene expression of *RAB2B* (**A**), *RAB3B* (**B**), *RAB9A* (**C**), *RAB11B* (**D**), *RAB27A* (**E**), *STX1A* (**F**) and *VAMP7* (**G**) with regards to the stage of the disease. No statistically significant association was observed between disease stage and relative expression of those genes. Figure S5. Relative gene expression of *RAB2B* (**A**), *RAB3B* (**B**), *RAB9A* (**C**), *RAB11B* (**D**), *RAB27A* (**E**), *STX1A* (**F**) and *VAMP7* (**G**) with regards to tumor grade. No statistically significant association was observed between tumor grade and relative expression of those genes. Figure S6. Relative gene expression of *RAB2B* (**A**), *RAB3B* (B), *RAB9A* (**C**), *RAB11B* (**D**), *RAB27A* (**E**), *STX1A* (**F**) and *VAMP7* (**G**) with regards to the N status from the TNM classification system. No statistically significant association was observed between the N status from the TNM classification system and relative expression of those genes. Figure S7. Relative gene expression of *RAB2B* (**A**), *RAB3B* (**B**), *RAB27A* (**C**), *STX1A* (**D**), and *VAMP7* (**E**), with regards to the presence of distant metastasis. No statistically significant association was observed between the presence of distant metastasis and relative expression of those genes. Figure S8. Relative gene expression of *RAB3B* (**A**), *RAB9A* (**B**), *RAB11B* (**C**), *RAB27A* (**D**), *RAB27B* (**E**), and *STX1A* (**F**) with regards to the T status from the TNM classification system. No statistically significant association was observed between the T status from the TNM classification system and relative expression of those genes. Figure S9. Kaplan Meier curves for 5-year overall survival of *RAB2B* (**A**), *RAB3B* (**B**), and *VAMP7* (**C**). No statistically significant differences are observed between 5-year survival and relative expression of those genes.

## References

[1] Kosaka N., Kogure A., Yamamoto T., Urabe F., Usuba W., Prieto-Vila M., Ochiya T. (2019). Exploiting the message from cancer: The diagnostic value of extracellular vesicles for clinical applications. Exp. Mol. Med..

[2] Baietti M.F., Zhang Z., Mortier E., Melchior A., Degeest G., Geeraerts A., Ivarsson Y., Depoortere F., Coomans C., Vermeiren E. (2012). Syndecan-syntenin-ALIX regulates the biogenesis of exosomes. Nat. Cell Biol..

[3] Dignat-George F., Boulanger C.M. (2011). The many faces of endothelial microparticles. Arterioscler. Thromb. Vasc. Biol..

[4] Klingeborn M., Stamer W.D., Bowes Rickman C. (2018). Polarized exosome release from the retinal pigmented epithelium. Adv. Exp. Med. Biol..

[5] Mashouri L., Yousefi H., Aref A.R., Ahadi A.M., Molaei F., Alahari S.K. (2019). Exosomes: Composition, biogenesis, and mechanisms in cancer metastasis and drug resistance. Mol. Cancer.

[6] Beach A., Zhang H.G., Ratajczak M.Z., Kakar S.S. (2014). Exosomes: An overview of biogenesis, composition and role in ovarian cancer. J. Ovarian Res..

[7] Logozzi M., Angelini D.F., Iessi E., Mizzoni D., Di Raimo R., Federici C., Lugini L., Borsellino G., Gentilucci A., Pierella F. (2017). Increased PSA expression on prostate cancer exosomes in in vitro condition and in cancer patients. Cancer Lett..

[8] Poteryaev D., Datta S., Ackema K., Zerial M., Spang A. (2010). Identification of the switch in early-to-late endosome transition. Cell.

[9] Théry C., Zitvogel L., Amigorena S. (2002). Exosomes: Composition, biogenesis and function. Nat. Rev. Immunol..

[10] Colombo M., Moita C., Van Niel G., Kowal J., Vigneron J., Benaroch P., Manel N., Moita L.F., Théry C., Raposo G. (2013). Analysis of ESCRT functions in exosome biogenesis, composition and secretion highlights the heterogeneity of extracellular vesicles. J. Cell Sci..

[11] Gao Y., Qin Y., Wan C., Sun Y., Meng J., Huang J., Hu Y., Jin H., Yang K. (2021). Small Extracellular Vesicles: A Novel Avenue for Cancer Management. Front. Oncol..

[12] Han Q.F., Li W.J., Hu K.S., Gao J., Zhai W.L., Yang J.H., Zhang S.J. (2022). Exosome biogenesis: Machinery, regulation, and therapeutic implications in cancer. Mol. Cancer.

[13] Umwali Y., Yue C.B., Zhang Y., Zhang X., Gabriel A.N.A. (2021). Roles of exosomes in diagnosis and treatment of colorectal cancer. World J. Clin. Cases.

[14] Xiao Y., Zhong J., Zhong B., Huang J., Jiang L., Jiang Y., Yuan J., Sun J., Dai L., Yang C. (2020). Exosomes as potential sources of biomarkers in colorectal cancer. Cancer Lett..

[15] Jang S.C., Kim O.Y., Yoon C.M., Choi D.S., Roh T.Y., Park J., Nilsson J., Lötvall J., Kim Y.K., Gho Y.S. (2013). Bioinspired exosome-mimetic nanovesicles for targeted delivery of chemotherapeutics to malignant tumors. ACS Nano.

[16] Tian Y., Li S., Song J., Ji T., Zhu M., Anderson G.J., Wei J., Nie G. (2014). A doxorubicin delivery platform using engineered natural membrane vesicle exosomes for targeted tumor therapy. Biomaterials.

[17] Tang Z., Li C., Kang B., Gao G., Li C., Zhang Z. (2017). GEPIA: A web server for cancer and normal gene expression profiling and interactive analyses. Nucleic Acids Res..

[18] Lánczky A., Győrffy B. (2021). Web-Based Survival Analysis Tool Tailored for Medical Research (KMplot): Development and Implementation. J. Med. Internet Res..

[19] Ramakers C., Ruijter J.M., Deprez R.H., Moorman A.F. (2003). Assumption-free analysis of quantitative real-time polymerase chain reaction (PCR) data. Neurosci. Lett..

[20] Sørby L.A., Andersen S.N., Bukholm I.R., Jacobsen M.B. (2010). Evaluation of suitable reference genes for normalization of real-time reverse transcription PCR analysis in colon cancer. J. Exp. Clin. Cancer Res..

[21] Dimitrakopoulos F.I.D., Antonacopoulou A.G., Kottorou A.E., Panagopoulos N., Kalofonou F., Sampsonas F., Scopa C., Kalofonou M., Koutras A., Makatsoris T. (2019). Expression of Intracellular Components of the NF-κB Alternative Pathway (NF-κB2, RelB, NIK and Bcl3) is Associated with Clinical Outcome of NSCLC Patients. Sci. Rep..

[22] Dimitrakopoulos F.I., Kottorou A.E., Rodgers K., Sherwood J.T., Koliou G.A., Lee B., Yang A., Brahmer J.R., Baylin S.B., Yang S.C. (2021). Clinical Significance of Plasma CD9-Positive Exosomes in HIV Seronegative and Seropositive Lung Cancer Patients. Cancers.

[23] Zhang W., Hu X., Jiang Z. (2022). Small Extracellular Vesicles: Key Forces Mediating the Development and Metastasis of Colorectal Cancer. Cells.

[24] Dong W., Cui J., Yang J., Li W., Wang S., Wang X., Li X., Lu Y., Xiao W. (2015). Decreased expression of Rab27A and Rab27B correlates with metastasis and poor prognosis in colorectal cancer. Discov. Med..

[25] Bao J., Ni Y., Qin H., Xu L., Ge Z., Zhan F., Zhu H., Zhao J., Zhou X., Tang X. (2014). Rab27b is a potential predictor for metastasis and prognosis in colorectal cancer. Gastroenterol. Res. Pract..

[26] Hua Y., Ma X., Liu X., Yuan X., Qin H., Zhang X. (2017). Identification of the potential biomarkers for the metastasis of rectal adenocarcinoma. Apmis.

[27] Cheng W.C., Liao T.T., Lin C.C., Yuan L.T.E., Lan H.Y., Lin H.H., Teng H.W., Chang H.C., Lin C.H., Yang C.Y. (2019). RAB27B-activated secretion of stem-like tumor exosomes delivers the biomarker microRNA-146a-5p, which promotes tumorigenesis and associates with an immunosuppressive tumor microenvironment in colorectal cancer. Int. J. Cancer.

[28] Shi C., Yang X., Ni Y., Hou N., Xu L., Zhan F., Zhu H., Xiong L., Chen P. (2015). High Rab27A expression indicates favorable prognosis in CRC. Diagn. Pathol..

[29] Huang Z., Feng Y. (2017). Exosomes derived from hypoxic colorectal cancer cells promote angiogenesis through Wnt4-Induced β-catenin signaling in endothelial cells. Oncol. Res..

[30] Feng F., Jiang Y., Lu H., Lu X., Wang S., Wang L., Wei M., Lu W., Du Z., Ye Z. (2016). Rab27A mediated by NF-κB promotes the stemness of colon cancer cells via up-regulation of cytokine secretion. Oncotarget.

[31] Blanc L., Vidal M. (2018). New insights into the function of Rab GTPases in the context of exosomal secretion. Small GTPases.

[32] Kobayashi M., Kawachi H., Hurtado C., Wielandt A.M., Ponce A., Karelovic S., Pasternak S., Delgado C., Pinto P., Carrasco H. (2018). A Pilot Trial to Quantify Plasma Exosomes in Colorectal Cancer Screening from the International Collaborative Study between Chile and Japan. Digestion.

