# Supplementary material for: Small Extracellular Vesicles (sEVs) Biogenesis Molecular Players Are Associated with Clinical Outcome of Colorectal Cancer Patients
Source: Cancers (Basel). 2023 Mar 9;15(6):1685. doi: 10.3390/cancers15061685 (PMC10046180; doi:10.3390/cancers15061685)
Supplement: Supplementary file 1 [file cancers-15-01685-s001.zip › cancers-2205083-supplementary.pdf]

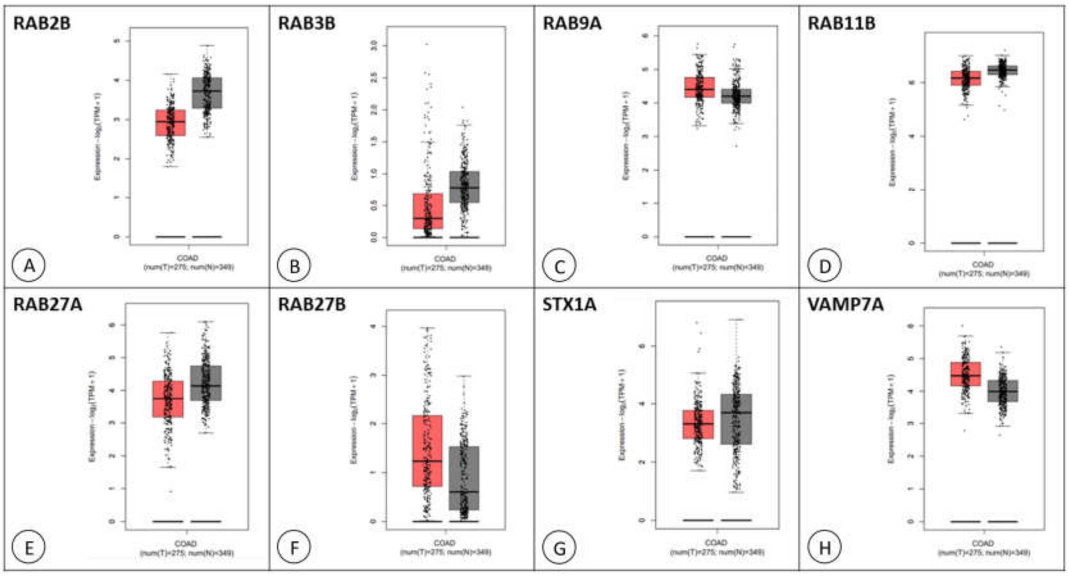

Supplementary figure S1. Dot-plots with superimposed box plots for the expression of 8 selected gene in colorectal cancer (red) and normal tissues (grey) using GEPIA web server. Box plots for the expression of (A) *RAB2B*, (B) *RAB3B*, (C) *RAB9A*, (D) *RAB11B*, (E) *RAB27A*, (F) *RAB27B*, (G) *STX1A* and (H) *VAMP7A*. Mean and standard deviation values were calculated for the comparison. Abbreviations: COAD, Colon adenocarcinoma; RAB2B, Ras-Related Protein Rab-2B; RAB3B, Ras-Related Protein Rab-3B; RAB9A, Ras-Related Protein Rab-9A; RAB11B, Ras-Related Protein Rab-11B; RAB27A, Ras-Related Protein Rab-27A; RAB27B, Ras-Related Protein Rab-28B; STX1A, Syntaxin 1A; VAMP7, Vesicle-Associated Membrane Protein 7.

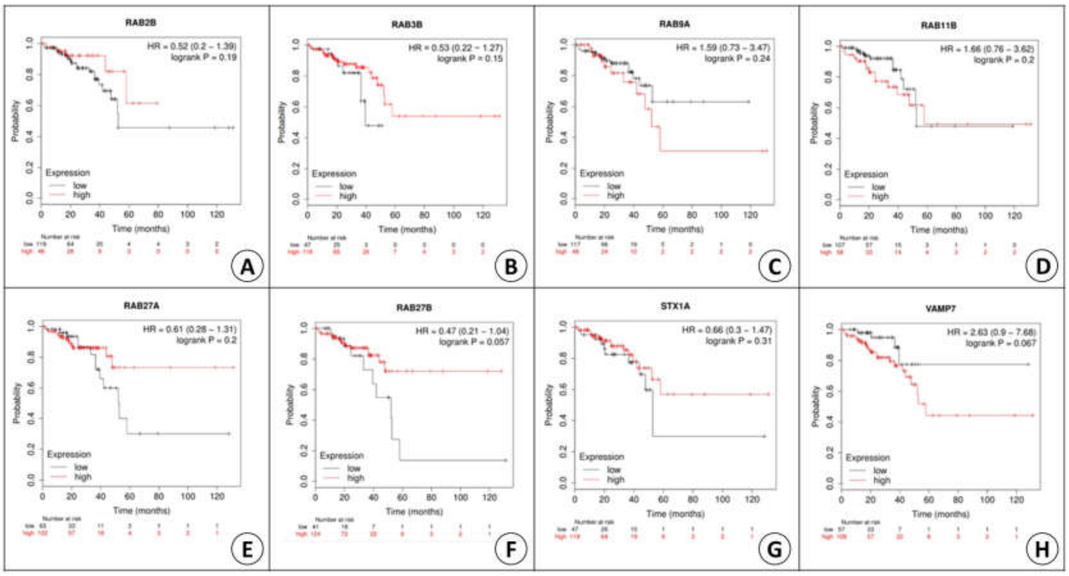

Supplementary figure S2. Kaplan Meier curves of overall survival using the "KMplotter" for the expression of (A) *RAB2B*, (B) *RAB3B*, (C) *RAB9A*, (D) *RAB11B*, (E) *RAB27A*, (F) *RAB27B*, (G) *STX1A* and (H) *VAMP7* in colorectal cancer patients using "KMplotter". Abbrevations: RAB2B, Ras-Related Protein Rab-2B; RAB3B, Ras-Related Protein Rab-3B; RAB9A, Ras-Related Protein Rab-9A; RAB11B, Ras-Related Protein Rab-11B; RAB27A, Ras-Related Protein Rab-27A; RAB27B, Ras-Related Protein Rab-28B; STX1A, Syntaxin 1A; VAMP7, Vesicle-Associated Membrane Protein 7.

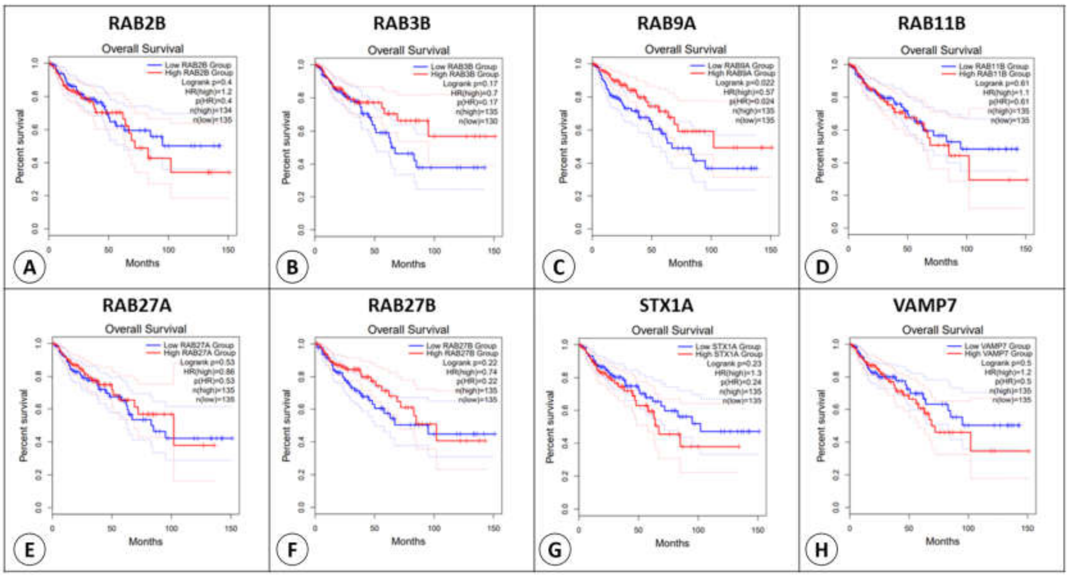

Supplementary figure S3. Kaplan Meir curves of overall survival for the expression of (A) *RAB2B*, (B) *RAB3B*, (C) *RAB9A*, (D) *RAB11B*, (E) *RAB27A*, (F) *RAB27B*, (G) *STX1A* and (H) *VAMP7* in colorectal cancer patients using the "GEPIA". Abbreviations: RAB2B, Ras-Related Protein Rab-2B; RAB3B, Ras-Related Protein Rab-3B; RAB9A, Ras-Related Protein Rab-9A; RAB11B, Ras-Related Protein Rab-11B; RAB27A, Ras-Related Protein Rab-27A; RAB27B, Ras-Related Protein Rab-28B; STX1A, Syntaxin 1A; VAMP7, Vesicle-Associated Membrane Protein 7.

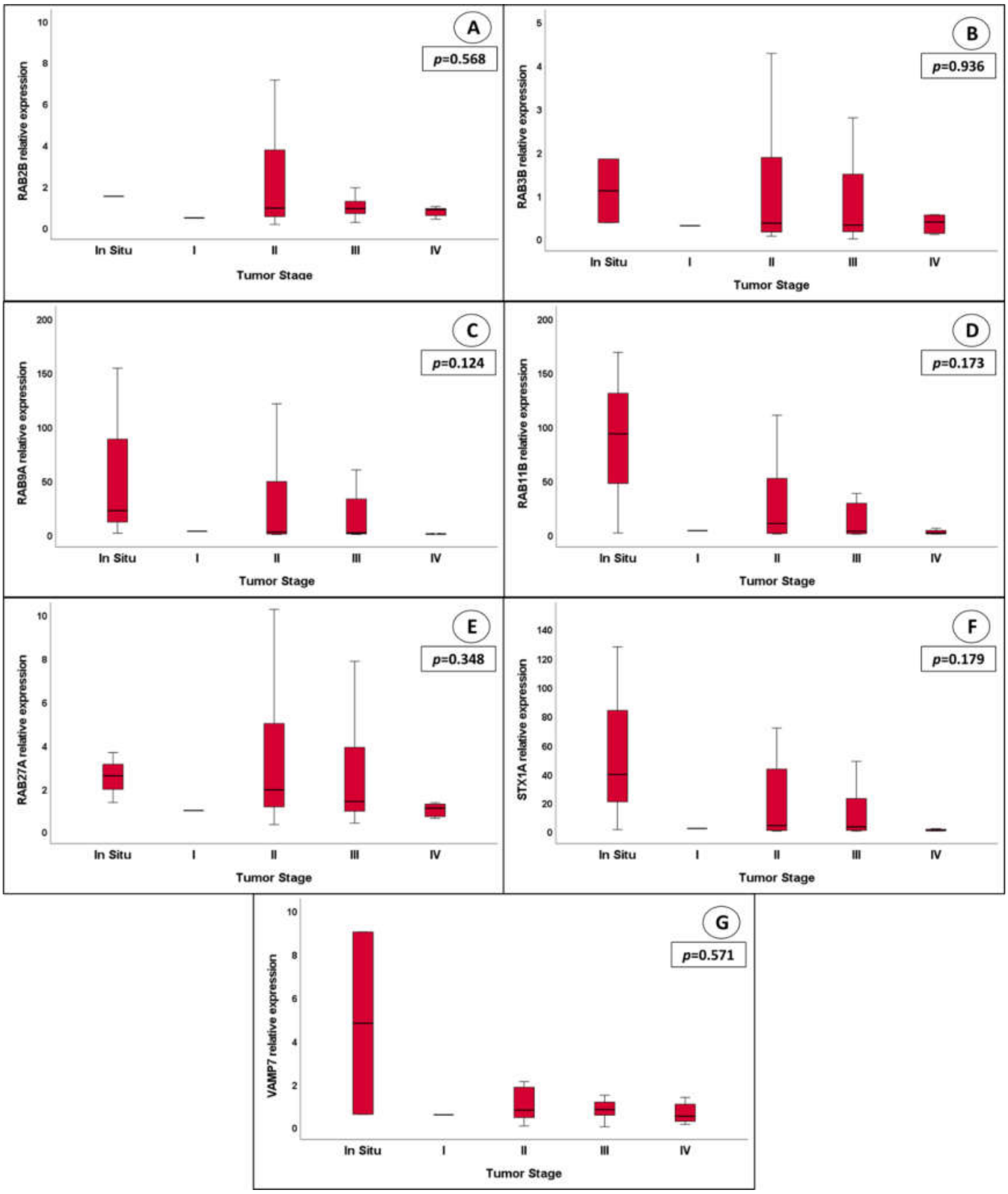

Supplementary figure S4. Relative gene expression of *RAB2B* (A), *RAB3B* (B), *RAB9A* (C), *RAB11B* (D), *RAB27A* (E), *STX1A* (F) and *VAMP7* (G) with regards to the stage of the disease. No statistically significant association was observed between disease stage and relative expression of those genes.

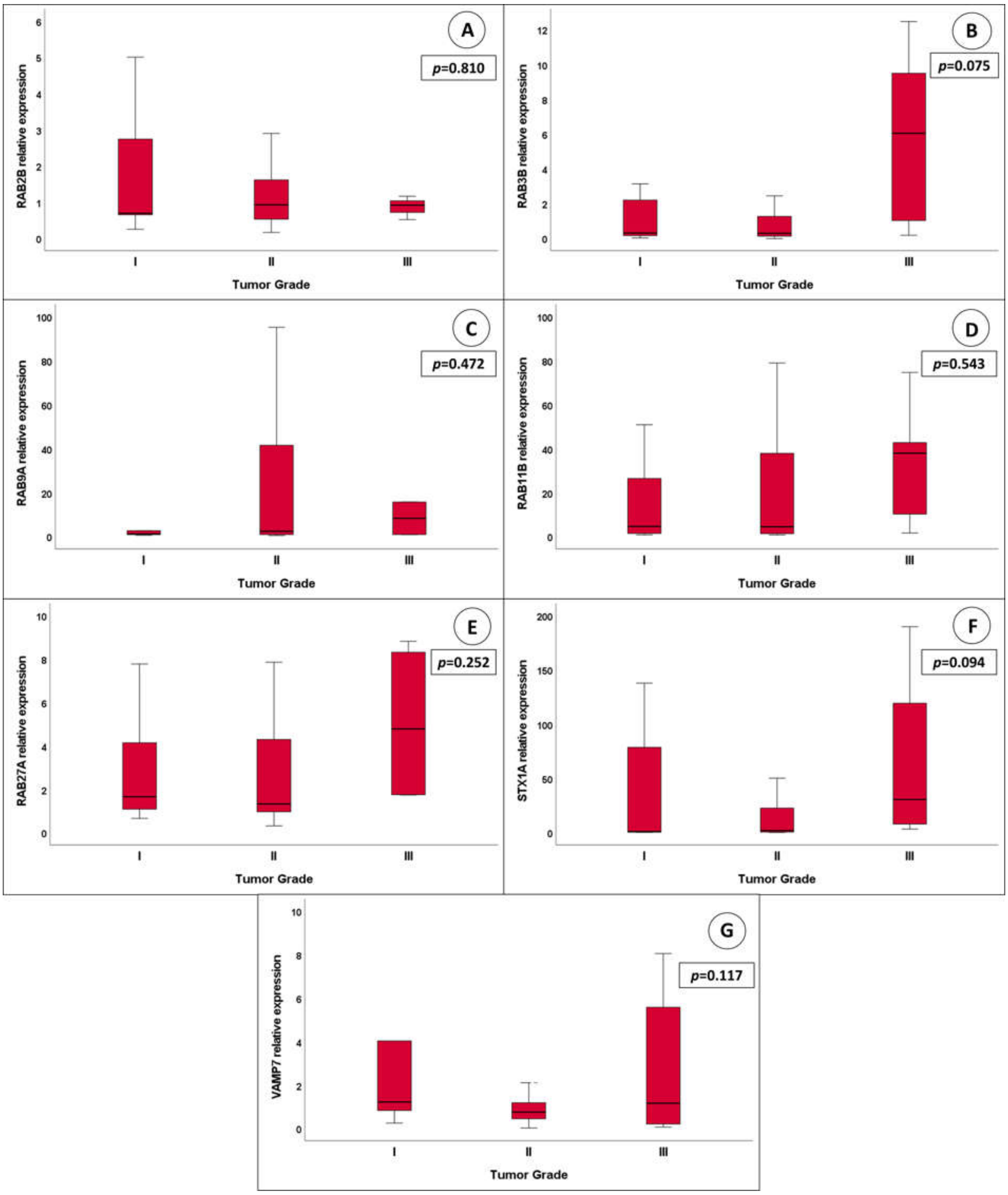

Supplementary figure S5. Relative gene expression of *RAB2B* (A), *RAB3B* (B), *RAB9A* (C), *RAB11B* (D), *RAB27A* (E), *STX1A* (F) and *VAMP7* (G) with regards to tumor grade. No statistically significant association was observed between tumor grade and relative expression of those genes.

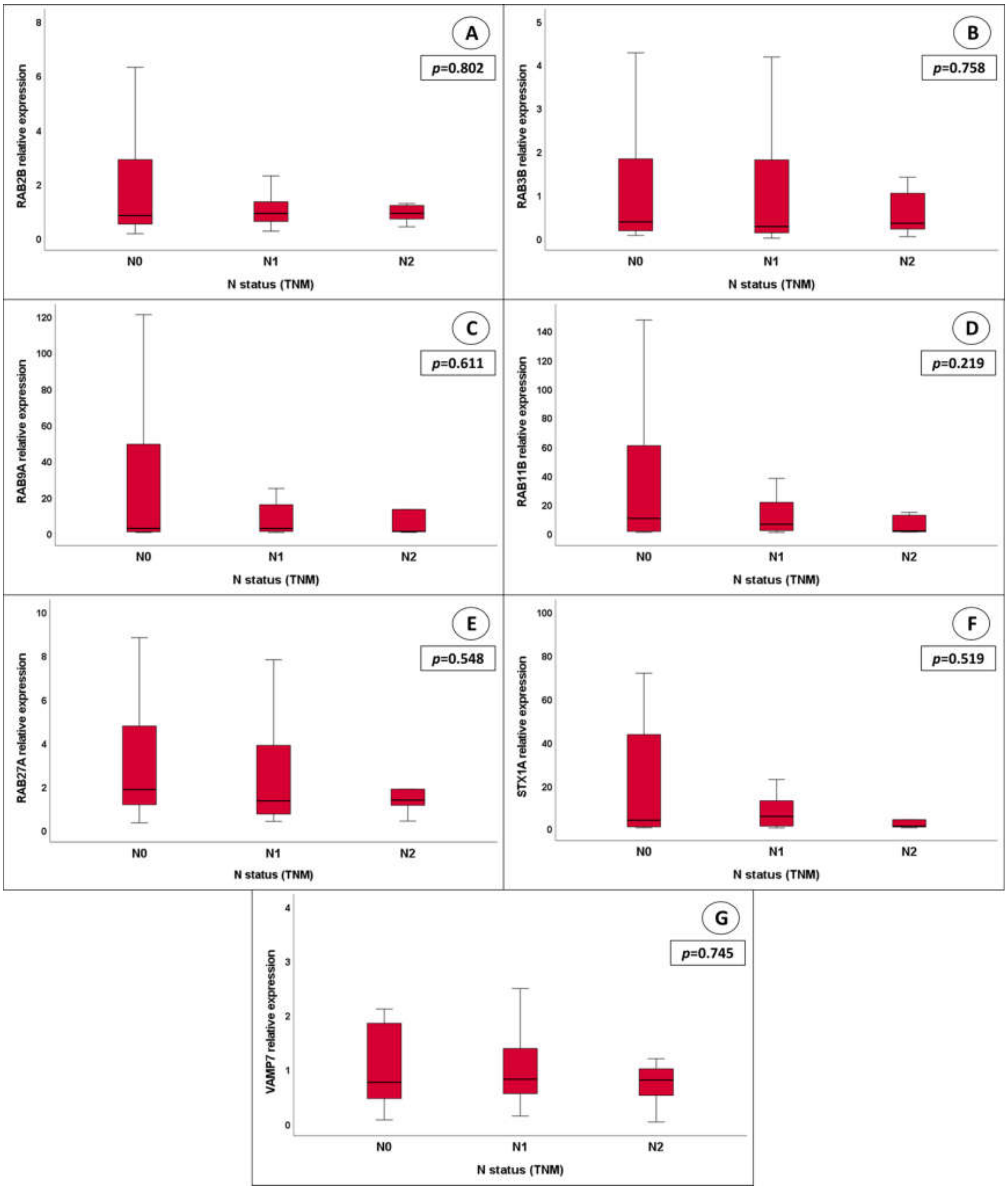

Supplementary figure S6. Relative gene expression of *RAB2B* (A), *RAB3B* (B), *RAB9A* (C), *RAB11B* (D), *RAB27A* (E), *STX1A* (F) and *VAMP7* (G) with regards to the N status from the TNM classification system. No statistically significant association was observed between the N status from the TNM classification system and relative expression of those genes.

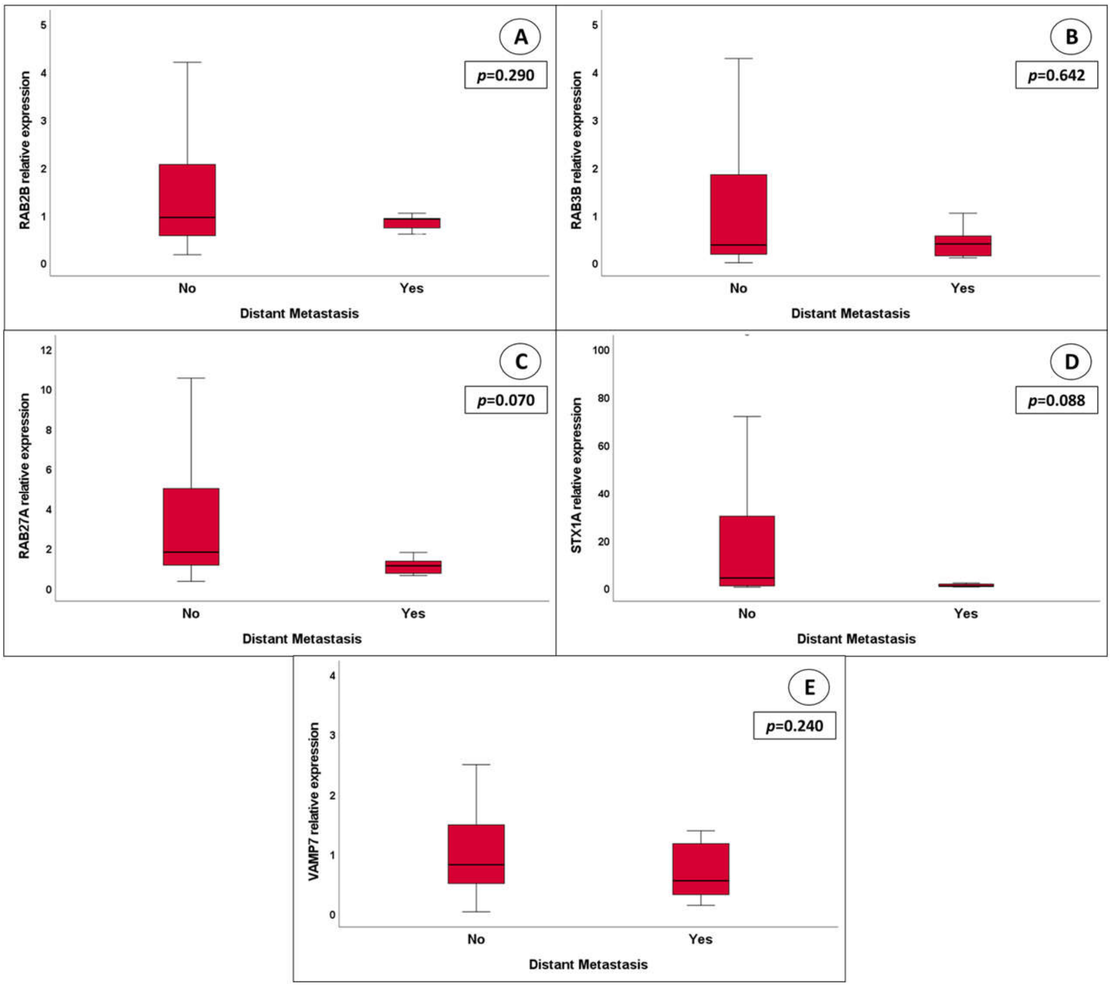

Supplementary figure S7. Relative gene expression of *RAB2B* (A), *RAB3B* (B), *RAB27A* (C), *STX1A* (D), and *VAMP7* (E), with regards to the presence of distant metastasis. No statistically significant association was observed between the presence of distant metastasis and relative expression of those genes.

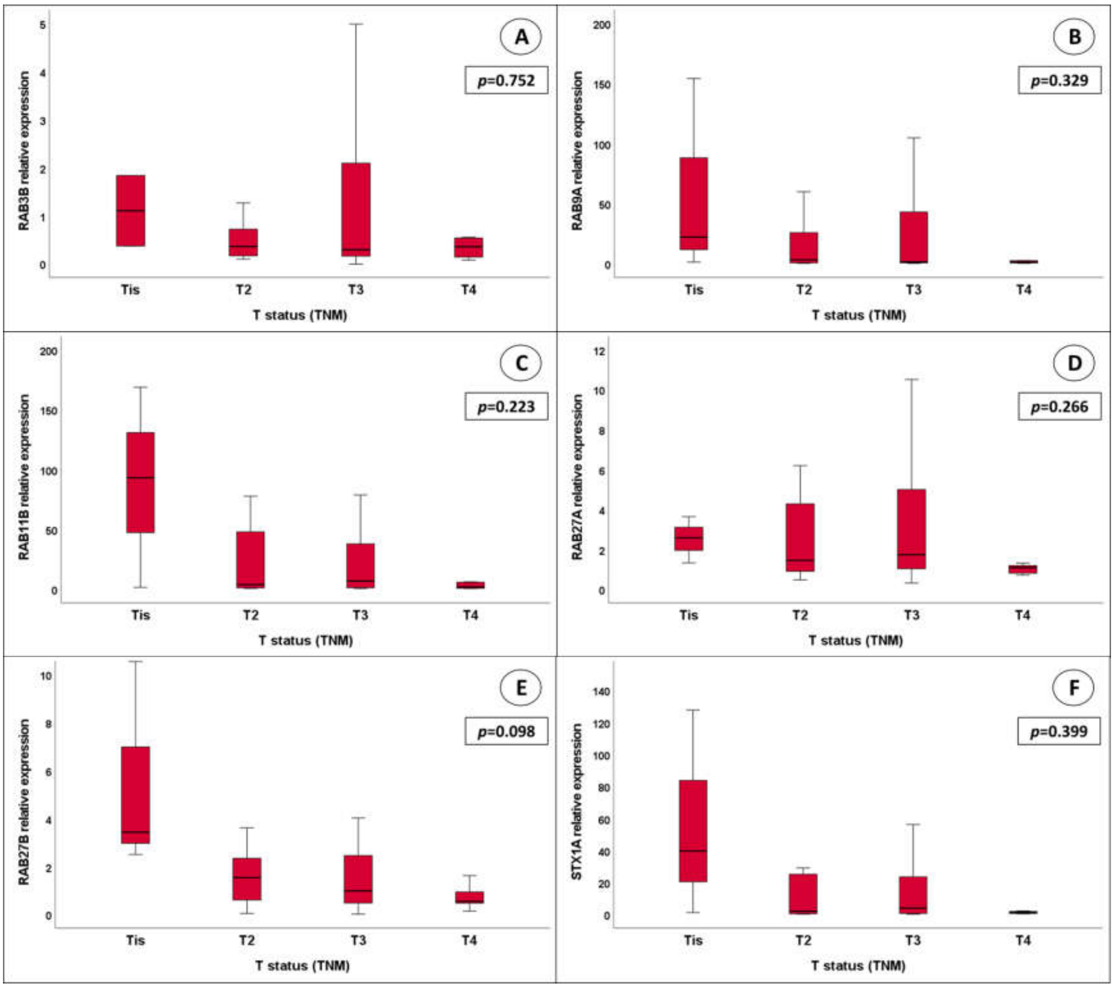

Supplementary figure S8. Relative gene expression of *RAB3B* (A), *RAB9A* (B), *RAB11B* (C), *RAB27A* (D), *RAB27B* (E), and *STX1A* (F) with regards to the T status from the TNM classification system. No statistically significant association was observed between the T status from the TNM classification system and relative expression of those genes.

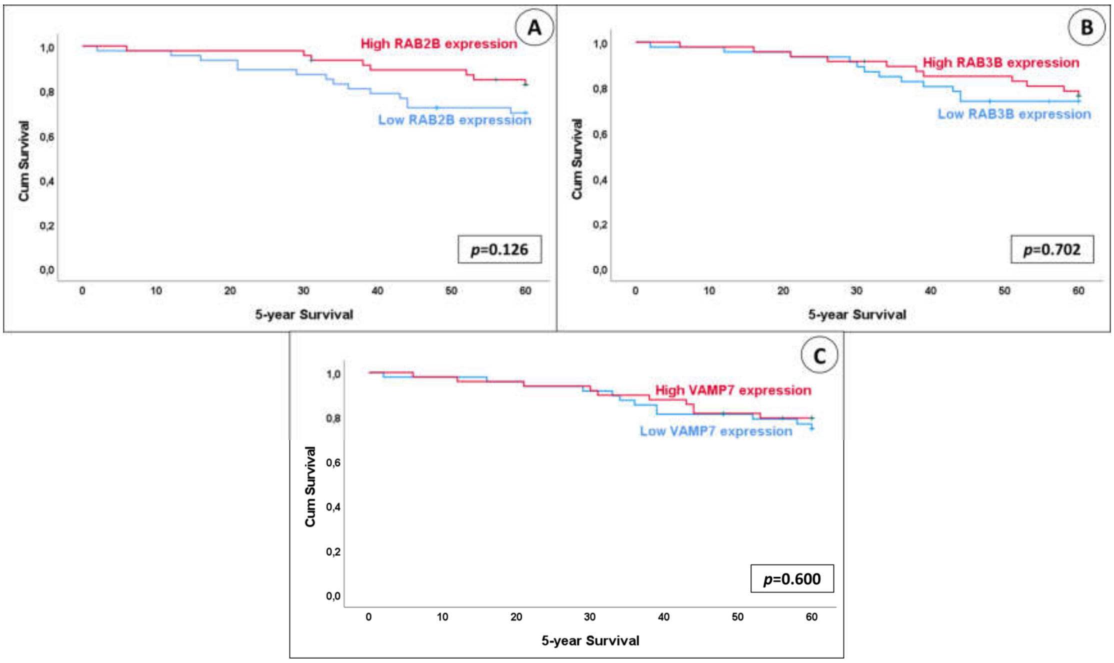

Supplementary figure S9. Kaplan Meier curves for 5-year overall survival of *RAB2B* (A), *RAB3B* (B), and *VAMP7* (C). No statistically significant differences are observed between 5-year survival and relative expression of those genes.
